# Quantifying Access Disparities in Response Plans

**DOI:** 10.1371/journal.pone.0146350

**Published:** 2016-01-15

**Authors:** Saratchandra Indrakanti, Armin R. Mikler, Martin O’Neill, Chetan Tiwari

**Affiliations:** Center for Computational Epidemiology and Response Analysis (CeCERA), University of North Texas, Denton, Texas, United States of America; Cardiff University, UNITED KINGDOM

## Abstract

Effective response planning and preparedness are critical to the health and well-being of communities in the face of biological emergencies. Response plans involving mass prophylaxis may seem feasible when considering the choice of dispensing points within a region, overall population density, and estimated traffic demands. However, the plan may fail to serve particular vulnerable subpopulations, resulting in access disparities during emergency response. For a response plan to be effective, sufficient mitigation resources must be made accessible to target populations within short, federally-mandated time frames. A major challenge in response plan design is to establish a balance between the allocation of available resources and the provision of equal access to PODs for all individuals in a given geographic region. Limitations on the availability, granularity, and currency of data to identify vulnerable populations further complicate the planning process. To address these challenges and limitations, data driven methods to quantify vulnerabilities in the context of response plans have been developed and are explored in this article.

## Introduction

The threat of bio-emergencies to the health and well being of communities has been widely recognized. In the United States, emergency preparedness has become one of the highest priorities of federal, state, and local governments [1] [2] [3]. The Pandemic and All-Hazards Preparedness Act (PAHPA) was signed into a law in 2006 and reauthorized (PAHPRA) in 2013 to improve the nation’s preparedness in the event of bio-emergencies [4]. This long-term initiative requires reorientation of the nation’s public health and health care infrastructure to reach, educate, and care for all citizens. The White House report on the federal response to Hurricane Katrina underscores the need for the Department of Health and Human Services to enhance its capabilities for public health and medical support during a crisis [5]. The Centers for Disease Control and Prevention (CDC) recommends and provides guidelines to public health administrators to develop emergency response plans by selecting suitable Points of Dispensing (POD) facilities in a geographic region and establishing their service areas to dispense prophylactic medications to populations in a timely manner [6]. Further, PAHPRA has broad implications for preparedness and response activities taking into account the needs of at-risk or vulnerable individuals.

Research focusing on the computational analysis and optimization of existing bio-emergency response plans has been gaining momentum due to the threat of adverse events, including the accidental or deliberate release of biochemical substances. A response plan is considered feasible only if available mitigation resources are sufficient to serve and are accessible to the target population within short, mandated time frames. A response plan generated using current methods [7] [8] may seem feasible when considering the spatial distribution of PODs within a given geographic region, overall population density, and estimated traffic demands. However, the plan may fail to serve particular vulnerable subpopulations, resulting in access disparities during a bio-emergency. The CDC and PAHPRA recognize that vulnerabilities in the population may serve as barriers placing certain individuals at-risk of not receiving critical medical resources [6]. Demographic indicators of vulnerability, such as lack of personal or public transportation and language barriers, have been identified by the CDC and in the PAHPRA [9]. Vulnerabilities stemming from the social, behavioral, cultural, economic, and health characteristics of subpopulations may induce the need for additional targeted resources in during bio-emergency response. In order to develop an effective bio-emergency response plan that minimizes access disparities for vulnerable subpopulations, the aggregate needs of such populations must be quantified. The inability to quantify these needs is a gap in critical bio-emergency response capabilities. Therefore, methodologies must be developed to quantify resource needs of vulnerable populations with respect to bio-emergencies. Once developed, these methodologies can be applied to minimize access disparities in bio-emergency response plans.

The assessment of vulnerabilities for a specific response plan necessitates the integration of data from disparate sources, which, when combined, will allow planners to identify geographic areas where response plan participation and access may be affected by specific vulnerabilities. A major challenge in response plan design is to establish a balance between the allocation of available resources and the provision of equal access to PODs for all individuals in a given geographic region. The large amount of data required for response plan design and analysis necessitates the application of computational tools to aid public health experts in preparing for bio-emergencies. To this end, the REPLAN Framework was designed to provide computational methods to facilitate the design, analysis and optimization of response plans for emergencies [10] [7]. The research into quantifying access disparities described in this article builds upon this framework enabling the quantification vulnerabilities in the context of specific response plans.

Vulnerabilities are classified into two different types according to the special resources needed to keep these vulnerabilities from leading to access disparities. Type-1 vulnerabilities involve changes in the placement (location) of infrastructural components, and Type-2 vulnerabilities require the adjustment of resource allocations to minimize access disparities. This article focuses on quantifying Type-1 and Type-2 vulnerabilities that can lead to access disparities during response plan implementation.

## Background and Related Work

Studies conducted to evaluate the preparedness of cities for mass dispensing of prophylactics have highlighted the challenges of such an operation [11] [12]. Multiple constraints such as time, population needs, and resource availability must be considered when building response plans. Disparate data sources may have to be utilized to retrieve and combine information pertaining to the various components of a response plan to accomplish this task. Public health experts need computational tools to effectively manage and use the large amounts of data required for data driven response plan design. Further, Geographic Information Systems (GIS) are needed to manage spatial and demographic information required to choose effective locations to host efficient dispensing points [13]. The significance of employing computational tools in solving public health problems has been well recognized [14]. Applications of computational tools to public health include computational systems such as the BioSense system [15] for public health surveillance created by the CDC that uses data from disparate sources to facilitate early detection of biological emergency events. Ensuring preparedness for public health emergencies requires computational frameworks for emergency response plan design.

There has been recent effort in the development of computational frameworks to aid in the design of response plans for public health emergencies. A simulation and decision support system called RealOpt was created to support planning and designing large-scale dispensing clinics for emergency response [16]. An emergency response decision support system to assist decision makers in evaluating and building emergency response plans, and selecting an appropriate one during an emergency event has been presented in [17]. RE-PLAN, a computational framework developed for regional public health departments to design and analyze response plans provides methods that assist in the optimal placement and design of PODs [7]. However data-driven tools and methods that enable the quantification of vulnerable populations in a response plan and facilitate the design of plans that provide uniform access to the concerned populations are yet to be developed.

The CDC has recognized demographic attributes which serve as indicators of vulnerability in populations during an emergency [9] [18]. The need for vulnerability analysis is noted in scientific literature [19]. Previous work on vulnerability focused on identifying places that needed attention to reduce the impact of hazards on populations specifically applicable to environmental, social, and economic systems [20] [21] [22]. The aim of vulnerability studies is to identify indicators of vulnerability and devise actions that will lead to the reduction of potential harm. Studies including [23] by Loucks et. al. identify critical social, economical, environmental and physical components that can be assessed by different indicators to understand the vulnerability of the system. Conceptual frameworks, such as the Risk-Hazard (RH) and the Pressure-and-Release (PAR) models that account for the vulnerability of coupled human-environment systems with diverse and complex linkages at multiple spatio-temporal scales have been developed [24] [25]. In order to develop an effective bio-emergency response plan that reduces access disparities for vulnerable subpopulations with unique characteristics, methodologies that facilitate the identification of such populations must be developed.

Access disparities in a response plan may be a consequence of the lack of means to reach a POD or the non availability of the required resources to address specific needs at a POD. The lack of means to reach a POD may be addressed by identifying vulnerable population groups and making public transit available to them. Several researchers have studied various aspects of accessibility of transit networks to the public. Los Alamos National Laboratory developed TRansportation ANalysis and SIMulation System (TRANSIMS) that simulates movements of the population across the transportation networks to support planning, traffic, and environmental research efforts [26]. Direct ridership models of rail and bus use combine walking distance estimates, transit service features, and geographic population and transit data to predict the usage of transit resources [27] [28]. The authors of [29] contrast the access and accessibility of a public transit system, and an extensive literature review regarding the concept of accessibility of public transit is provided in [30]. Block group level data was used in [31] to create maps of health indicators related to transit network characteristics such as walkability and accessibility. The effect of lack of access to public transportation in receiving services has been highlighted in works including [32], a study of cancer patients in New Mexico which revealed impaired access to transportation as being a significant factor in non-receipt of cancer therapies.

Certain vulnerable sub-populations require special resources during an emergency to address their specific needs. To better serve the public during an emergency, emergency responders must be equipped with knowledge about the needs of various at-risk populations in the community. The challenges of studying populations and identifying vulnerabilities has been discussed in [33]. A variety of tools and methods have been developed to identify vulnerable sub-populations. Saliba et. al. developed a tool to identify vulnerable elderly people in a community [34]. Work by Phillips et. al. in [35] gives an overview of the state of social science research specific to populations at risk in the context of weather forecasting and warnings. The CDC provided guidelines to local authorities to define, locate and reach special, vulnerable, and at-risk populations in an emergency [9]. Increase the effectiveness of response plans [36] requires incorporating available skills and resources, domain knowledge, and communication strategies [37] [38] into response plan design. Computational methods that identify vulnerable population subgroups whose access to services at PODs during an emergency is restricted due to special needs, help enhance response plan reach and efficiency.

## Access Disparities and Vulnerability

In the context of this work, the definition of vulnerability is adopted from [19], which describes vulnerabilities as “the pre-event inherent characteristics or qualities of social systems that create potential barriers increasing the likelihood of inability to receive appropriate communication or required services” during an emergency event. Vulnerable individuals are those who have, in addition to their medical needs, other needs that may interfere with their ability to access or receive needed care during an emergency. When the special needs of vulnerable individuals are not sufficiently addressed, the response plan may fail to serve particular sub-populations, consequently resulting in *access disparities* during the emergency. Minimizing access disparities in a response plan is critical to the effectiveness of the plan.

PAHPRA identified various population subgroups including children, senior citizens, and pregnant women as being vulnerable in the event of an emergency. Individuals who may need additional response assistance also include those who have disabilities; live in institutionalized settings; are from diverse cultures; have limited English proficiency or are non-English speaking; are transportation disadvantaged; have chronic medical disorders; and have pharmacological dependencies. In the context of public health emergencies, CDC recognizes language, literacy, medical conditions and disabilities (physical, mental, cognitive, or sensory), isolation (cultural, geographic, or social), and age as major indicators of vulnerability, which may impede access to the assigned PODs during an emergency [9].

The access disparities in response plans arising as a consequence of unaccommodated vulnerabilities can be minimized by either introducing spatial modifications involving POD facility locations and their service area boundaries or by adjusting allocation of resources in the response plan. Depending on the means employed to address the vulnerabilities, we define two types of vulnerabilities: Type-1 vulnerabilities that involve changes in the placement (location) of infrastructural components; Type-2 vulnerabilities that require adjustment of resource allocation. Lack of access to private transportation and limited mobility are examples of Type-1 vulnerabilities that may be addressed by relocating service facilities such as PODs or public transportation access points so as to reduce the maximum permissible or acceptable walking distance thereby increasing the level of participation. Language barriers and physical disabilities (immobility due to age or health) exemplify Type-2 vulnerabilities that may require the assignment or reallocation of translators or special care personnel to the service facilities to extend the reach of a specific response plan.

### Quantification of Vulnerability

A response plan for a region includes locations of POD facilities and the respective sub-regions served by the PODs referred to as service area. The location and design of POD facilities are established with respect to the population composition of the sub-regions served by the PODs. A response plan *R* for a region can be represented as a one-one mapping *R* = <*P*, *S*>, where *P* is the set of PODs and *S* is the set of service areas which are non-overlapping sub-regions.

Devising strategies to minimize disparities in access to PODs during an emergency requires quantifying vulnerabilities in existing response plans. Quantification of Type-1 and Type-2 vulnerabilities identifies populations whose needs cannot be addressed under the current response plans and facilitates estimating the resource and infrastructural demand imposed on PODs due to vulnerable populations. In this section, we describe the quantification of Type-1 and Type-2 vulnerabilities in POD-based response plans.

#### Type-1 Quantification

**Fig 1:**
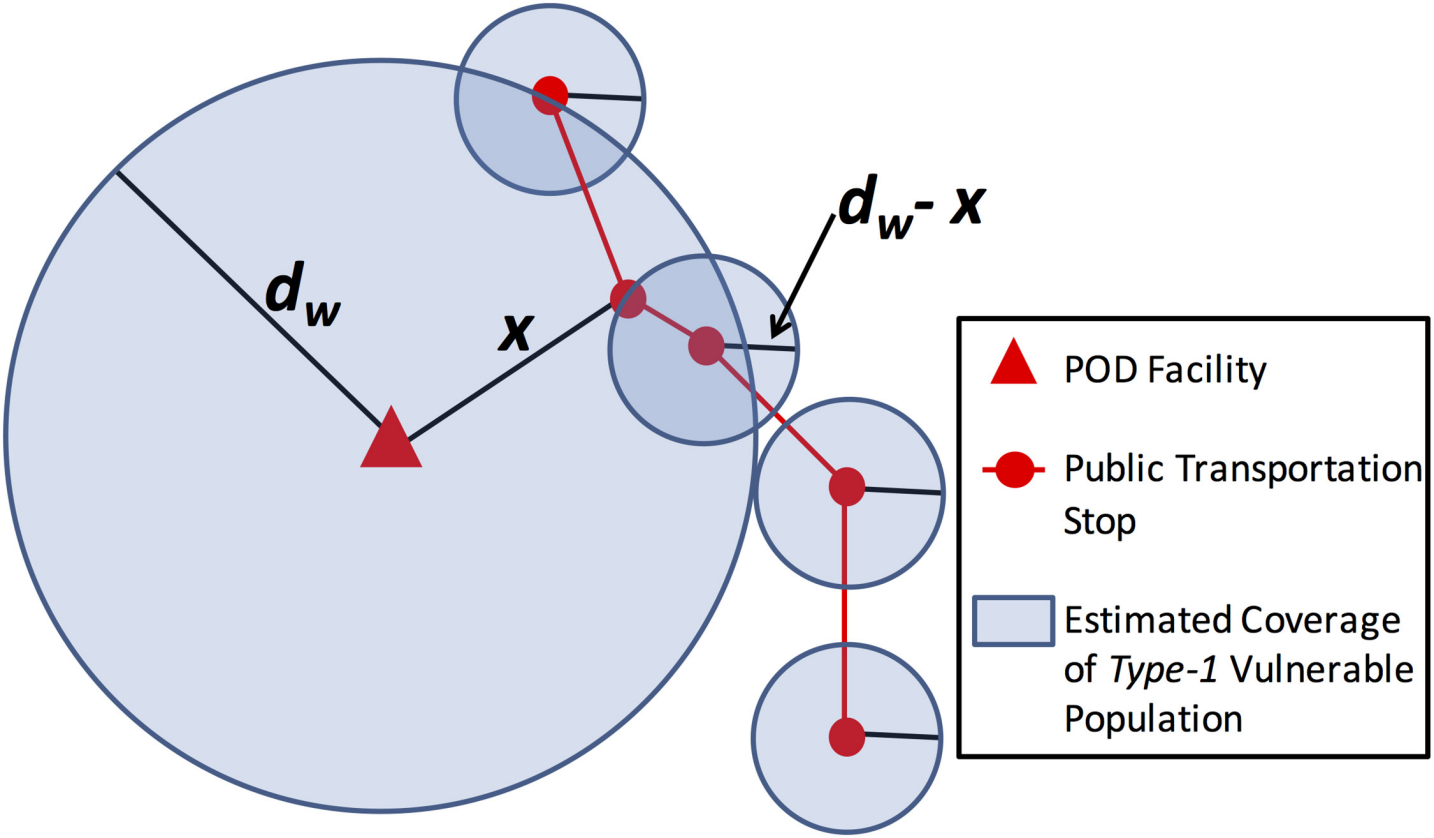
Type-1 Quantification: Coverage area estimation. Estimating the coverage area of POD for Type-1 transportation vulnerable population.

Lack of access to private transportation exemplifies Type-1 vulnerabilities which may require changes in the placement of infrastructural components. The spatial distribution of Type-1 vulnerabilities resulting from lack of access to private transportation are assessed with respect to the placement of each POD *p* in the set of PODs *P* of the existing response plans. The coverage area of existing response plans, given specific POD locations, is estimated on the basis of the definitions of maximum walking distance, *d*_*w*_. Multiple definitions of *d*_*w*_ can be used to represent physical limitations of different demographic groups or differences in the walkabiliity [30] over the region. A plan’s reach of vulnerable populations is calculated by overlaying the coverage area and the spatial distribution of vulnerable populations as exemplified in Fig 1.

**Fig 2:**
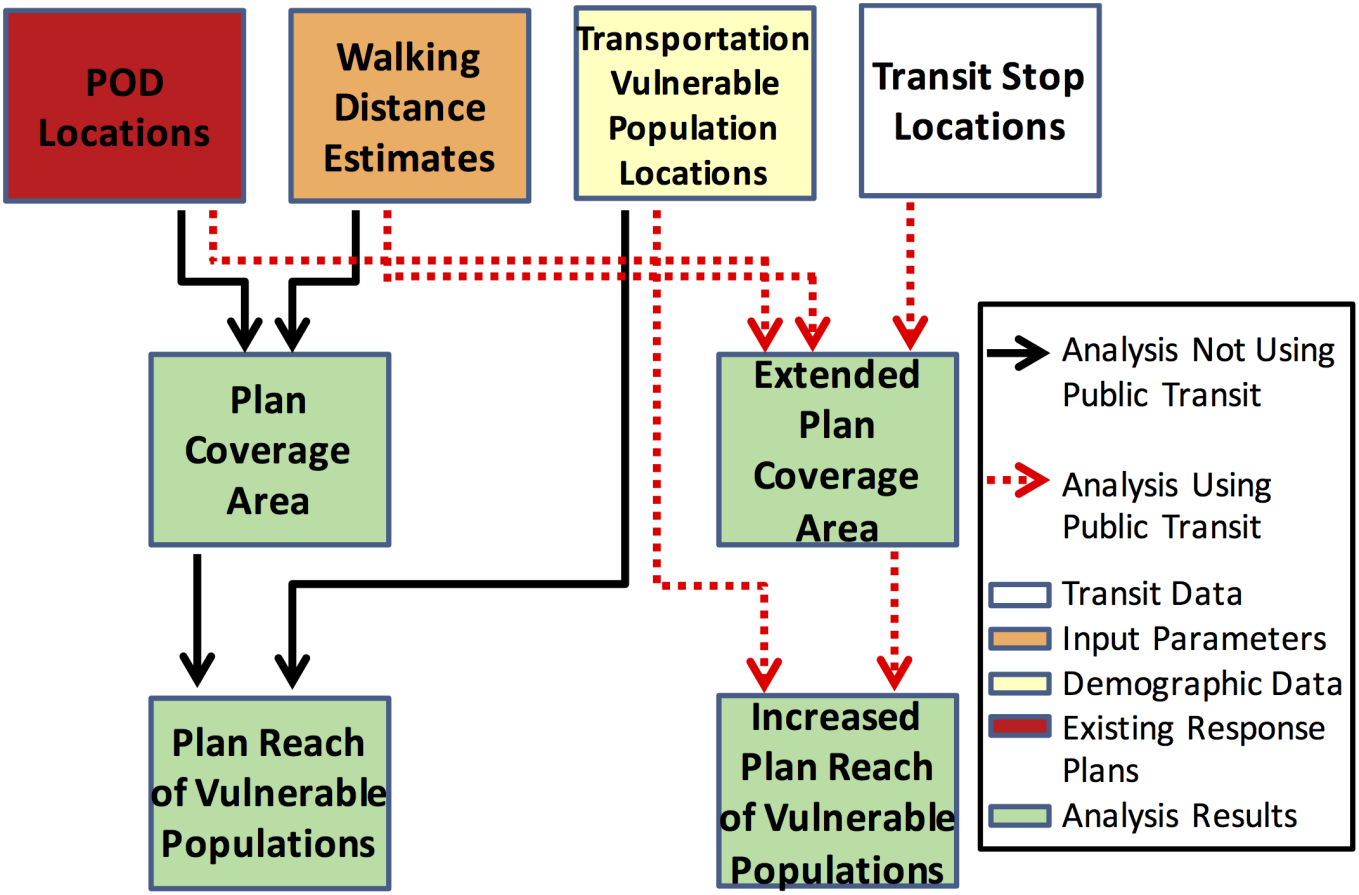
Type-1 Quantification: Summary. Summary of Type-1 transportation vulnerability quantification and analysis.

Public transit is used in conjunction with existing POD locations and estimations of acceptable walking distances to extend the reach of PODs in existing response plans. The effect of public transportation on access disparities is analyzed using local General Transit Feed (GTF) data, which consists of transit routes, schedules, and stop locations. The GTF specification was originally created by developers at Google and is used by such tools as Google Maps and Bing Maps to automatically provide public transit directions to mobile and online users. Existing response plans include a set of PODs *P*, and the public transit system includes a set of stops *S*. The distance between a POD *p*_*i*_ and a stop *s*_*j*_ is *d*(*p*_*i*_, *s*_*j*_). For all *p*_*i*_ ∈ *P* and *s*_*j*_ ∈ *S*, if *d*(*p*_*i*_, *s*_*j*_)<*d*_*w*_, then the transit stop *s*_*j*_ may be used to extend the walking distance coverage of POD *p*_*i*_. Increased reach of vulnerable populations resulting from the extension of walking distance coverage would then be quantified. The coverage area *C*_*p*_*i*__ of a POD *p*_*i*_ may be expanded from $Cp_{i}=d_{w}^{2}\pi$ up to $C_{p_{i}}=d_{w}^{2}\pi +{\left(d_{w}-x\right)}^{2}\left(|S|-1\right)\pi$ as shown in Fig 1. A summary of how a plan’s reach of vulnerable populations are calculated from input data is depicted in Fig 2. Thus, deficiencies of existing response plans with respect to specific vulnerable populations can be identified.

#### Type-2 Quantification

Type-2 vulnerabilities include vulnerabilities such as language barriers that can be addressed by the appropriate assignment of resources at PODs. Resources such as personnel or medical equipment that address specific Type-2 vulnerabilities must be strategically allocated to PODs to minimize access disparities in the response plan. To estimate the resource demand at a POD, the vulnerability at the POD is first quantified by computing the numbers of individuals susceptible to that vulnerability within its service area. The service area of a POD is a collection of population blocks such as census blocks, which are geographic entities with representative population and vulnerability data. POD specific lists of quantification of vulnerabilities are computed for the set of Type-2 vulnerabilities of interest. The lists generated are used to estimate the resource requirement at each POD.

Type-2 vulnerabilities, such as inability to speak in English, or physical disabilities, represented by the list *V* = *v*_1_, *v*_2_, …, *v*_|*V*|_ are analyzed using indicators recognized by the CDC and PAHPRA. For each POD *p*, a list of vulnerable populations *Q*(*p*) = *q*_*p*,1_, *q*_*p*,2_, …, *q*_*p*,|*V*|_ corresponding to the list of vulnerabilities *V* is calculated. *q*_*p*, *i*_, the quantification of vulnerability *v*_*i*_ at a POD *p* is calculated by aggregating the numbers of vulnerable individuals in each population block *b* ∈ *B*_*p*_, where *B*_*p*_ is the set of population blocks serviced by the POD *p*. The quantification of vulnerable populations *Q* having Type-2 vulnerabilities *V* is used to estimate specific resource needs across the geographic region in the response plan.

Addressing access disparities resulting as a consequence of Type-2 vulnerabilities requires optimal allocation of resources at PODs. In this work, resources are defined as specific items or personnel that assist the special needs of vulnerable populations at PODs during an emergency. Resources may refer to medical care resources including physical items such as surgical masks, vaccines or personnel such as physicians, nurses and translators. A list of resources *R* = *r*_1_, *r*_2_, …, *r*_|*R*|_ represents the resources available to address the vulnerabilities *V*. For each resource type *r* ∈ *R*, list *U*(*r*) = *u*_*r*,1_, *u*_*r*,2_,.., *u*_*r*, *m*_ denotes the units of *r* available during the emergency. A mapping*W* < *v*_*i*_, *r*_*a*_, *w*> indicates that the number *w* of individuals with vulnerability *v*_*i*_ addressed by one unit of the corresponding resource type *r*_*a*_. The resource demand at a POD *p* is given by the list *D*(*p*) = *γ*_*p*,1_, *γ*_*p*,2_,.., *γ*_*p*,|*V*|_ where *γ*_*p*, *i*_, the resource demand arising at POD *p* due to vulnerability *v*_*i*_ is calculated as $\gamma_{p,i}=\frac{q_{p,i}}{w}$.

The quantity of resources allocated at each POD *p* is given by the list *A*(*p*) = *a*_*p*,1_, *a*_*p*,2_,.., *a*_*p*,|*V*|_ where *a*_*p*, *i*_ is the quantity of resource allocated to POD *p* to address the vulnerability *v*_*i*_. In order to minimize the number of vulnerable individuals whose needs cannot be addressed, an allocation of resources to PODs represented by the mapping *S* < *u*_*i*, *j*_, *p* > ∀*u*_*i*, *j*_ ∈ *U*(*r*_*i*_), *r*_*i*_ ∈ *R* where *u*_*i*, *j*_ is a unit of resource of type *r*_*i*_ allocated to POD *p* ∈ *P*, the following expression is minimized:

$$\begin{array}{c} Minimize\sum_{i=1}^{\left|P\right|}\sum_{j=1}^{m}\left|\gamma_{i,j}-a_{i,j}\right| \end{array}$$

The computation of resource demand at PODs for each vulnerability leads to the following three cases to consider for resource allocation, based on the net resource availability.

**Case 1** The net availability of a resource type *r*_*a*_ such as translators, given by *N*(*r*_*a*_) is sufficient to meet the demand for it at all PODs. The availability of the resource is adequate to meet (i) the net demand at PODs, i.e., *N*(*r*_*a*_) > ∑_*p*_*i*_ ∈ *P*_ *γ*_*ia*_ and (ii) net regional requirement, i.e., *N*(*r*_*a*_) > ∑_*p*_*i*_ ∈ *P*_ *w* ⋅ *v*_*ij*_, where *w* is the quantity of vulnerability *v*_*j*_ addressed by a unit of resource *r*_*a*_.

**Case 2** Sufficient resources are available to meet the total regional demand for a resource type *r*_*a*_, however this does not meet the aggregate demand from all PODs, ∑_*p*_*i*_ ∈ *P*_ *γ*_*ia*_. This is represented by *N*(*r*_*a*_) < ∑_*p*_*i*_ ∈ *P*_ *γ*_*ia*_ and *N*(*r*_*a*_) > ∑_*p*_*i*_ ∈ *P*_ *w* ⋅ *v*_*ij*_. For instance, when the population of a region as a whole is considered, there could be a requirement for 100 units of a resource. However, the demand at individuals PODs could sum up to 120 units, which could result in a shortage of resources when the requirement at the regional level is considered.

**Case 3** The net availability of resource *r*_*a*_, *N*(*r*_*a*_) < ∑_*p*_*i*_ ∈ *P*_ *γ*_*ia*_, and *N*(*r*_*a*_) < ∑_*p*_*i*_ ∈ *P*_ *w* ⋅ *v*_*ij*_. In this case the resource is scarce and the demand at all PODs cannot be met.

**Fig 3:**
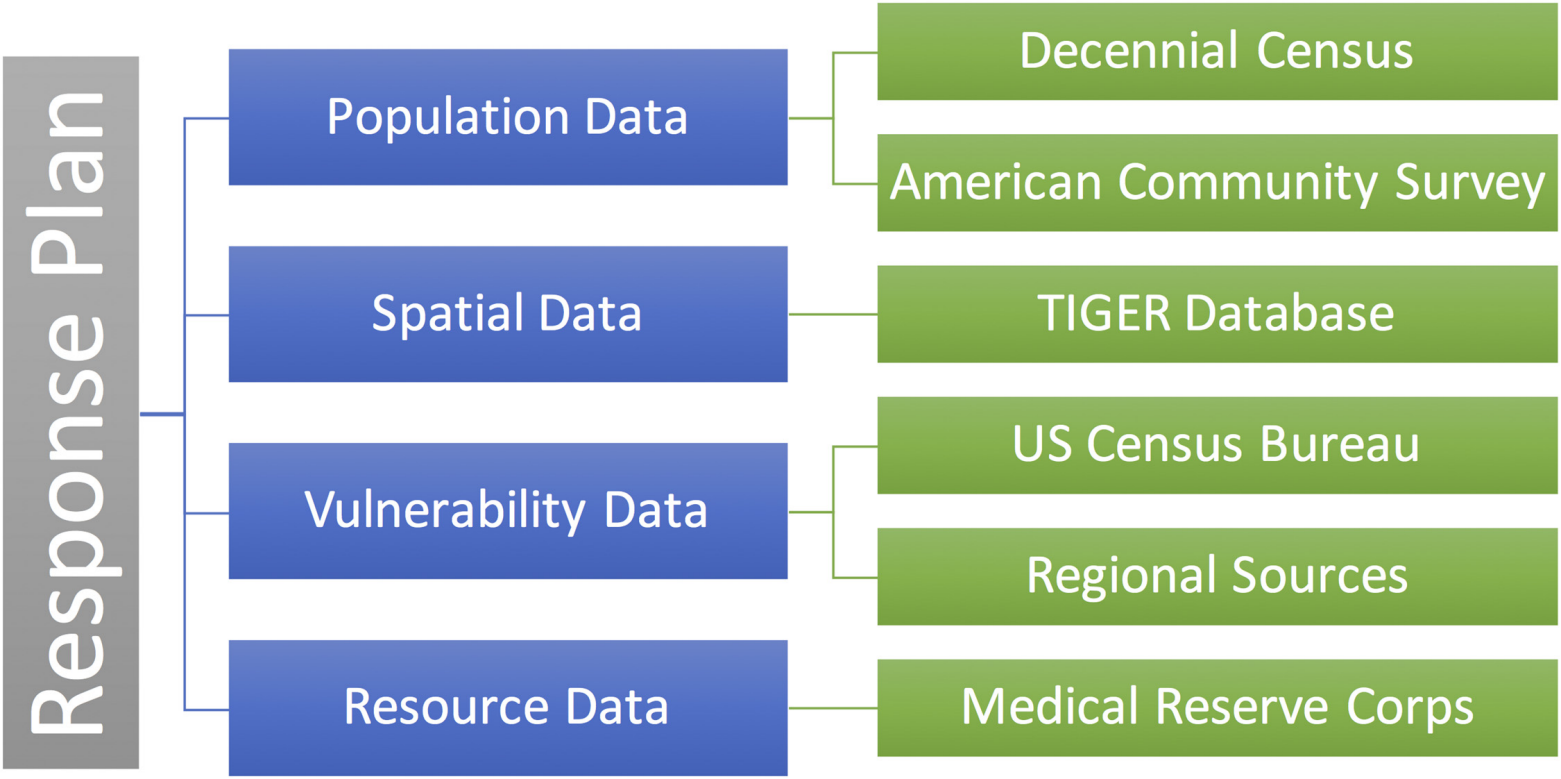
Data Management. Overview of the different data sources utilized and their interplay.

The design of response plans is driven by the utilization of diverse region-specific data procured from multiple sources. This may include different types of data such as population data, spatial data, or data pertaining to vulnerabilities or resources. Fig 3 depicts the different types of data involved in the design of response plans that address needs of vulnerable populations. Population data made available by sources such as the US Census Bureau through the decennial census or the American Community Survey (ACS) provide population counts within individual population blocks of the region. The population data, in conjunction with spatial data corresponding to the geography of the region is utilized in establishing partitioning of the region into service areas for PODs. Vulnerability data, consisting of numbers of vulnerable individuals within population blocks are used to quantify the vulnerabilities associated with each POD. Type-1 and Type-2 vulnerability data is obtained from data sources such as ACS, decennial census, or regional data sources. The presence of access disparities is computed based on the availability of vulnerability-specific resources at PODs. Resource data, including data from transit feeds or volunteer data from sources such as the Medical Reserve Corps (MRC), may be incorporated into existing response plans to obtain plans that address vulnerabilities.

The different data sources differ from each other in terms of data granularity, currency and attributes provided. Ensuring consistency with respect to spatial granularity of data and time of release across data sources is of importance. Since the features of a geographic region such as boundaries are subject to change, and the associated population counts may vary due to the change in boundaries or change in regional population with time, the underlying data obtained from different sources must correspond to approximately the same time period. Hence, different types of data employed in designing a particular response plan are ensured to be representing a similar time period. For instance, when population or vulnerability data is obtained from ACS 2009–2013, shapefiles from this period that provide spatial data of the region are used. Further, it is ensured that all the types of data used represent the same level of spatial granularity. Detailed data for population and spatial data for a region may be available at the census block level. However, data pertaining to certain vulnerabilities may only be available at a coarser level of granularity such as census tracts. Although data of a finer granularity can lead to more accurate planning, the non-availability of certain types of data at the finest granularity suggests maintaining consistency across sources to ensure correctness of plans. Data from sources such as the decennial census may offer a finer spatial granularity than others such as the ACS. However, the coarser data from ACS may be more current than the decennial census. While, data from multiple sources provide a tradeoff between spatial granularity and currency of data, it is important to ensure consistency among the selected data. In this work, response plans are designed using data from ACS 2009–2013 at the census tract level, the level of granularity that is satisfied by all of the underlying types of data.

**Fig 4:**
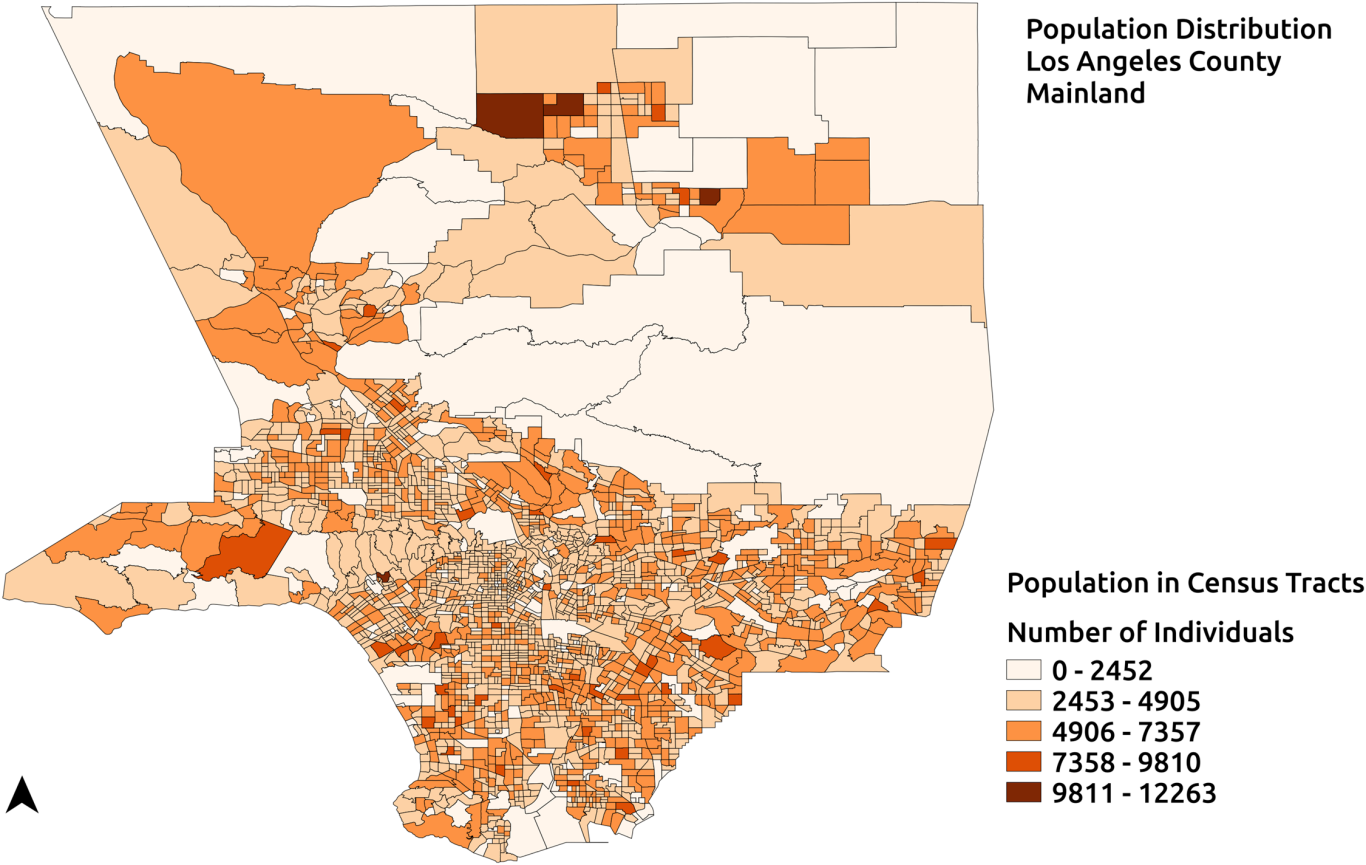
Population Distribution. Population distribution of the region representing the mainland of Los Angeles county.

**Fig 5:**
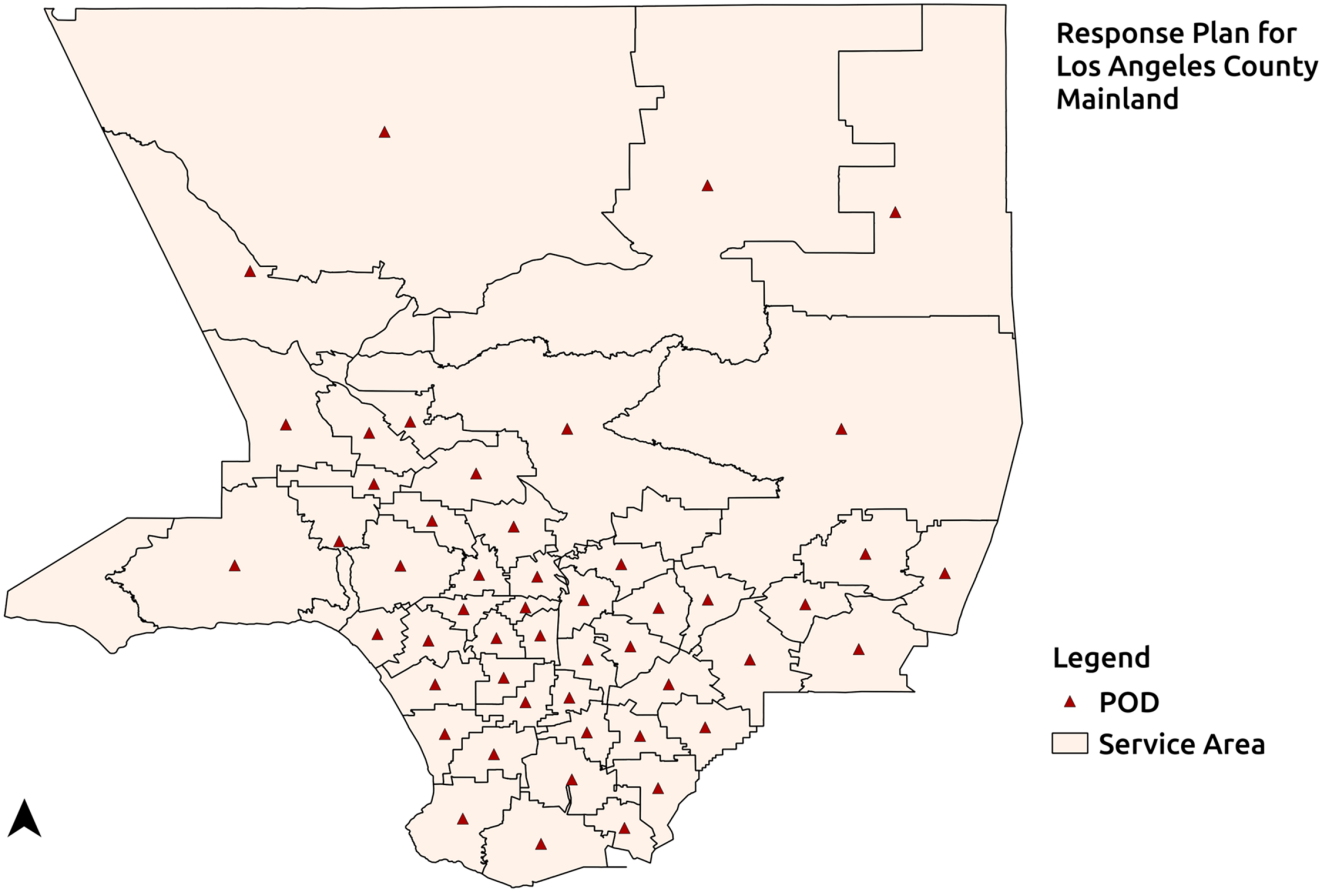
Response Plan. 50 Equal-population service areas generated for this region and their corresponding POD locations.

In this section the quantification of Type-1 and Type-2 vulnerabilities is exemplified using the region representing the mainland of Los Angeles County, California shown in Fig 4. Spatial data for this region is obtained from 2010 Tiger/Line shapefiles for Los Angeles County. Population counts and language vulnerability data at the census tract level are obtained from American Community Survey 2009–2013. The transit feeds used in the quantification of Type-1 transportation vulnerability are provided by Los Angeles County Metropolitan Transportation Authority and obtained from GTFS data exchange, a website that helps developers and transit agencies efficiently share and retrieve GTF data [39]. Fig 4 shows the population distribution of the mainland of Los Angeles County, California at the census tract level. This region, which has a population of 9893481 is partitioned into 50 service areas using the Equal Population partitioning(EPP) method [8]. In accordance with CDC recommendations about uniformity of PODs [36], EPP method implemented in the RE-PLAN framework partitions a given geographic region into equal population sub-regions. These equally populated partitions represent equally populated service areas that can adopt a uniform POD design. Potential service areas generated by EPP for the region of study based on population data at the census tract level can be seen in Fig 5 with the sub-regions enclosed by the solid lines representing equal population service areas and corresponding red triangles representing their PODs. For the purpose of illustration, PODs are placed at the geographic centroids of the potential service areas.

### Type-1 Results

The methods to quantify Type-1 vulnerabilities in existing response plans described previously are employed on the response plan for the region representing the mainland of Los Angeles county with 50 service areas generated by EPP. Transportation vulnerability in the response plan is quantified both with and without including the transit network for the region. First, the number of individuals without access to private transportation in the response plan is computed. Next, the number of individuals among the transportation vulnerable ones who have access to PODs when the transit network for the region is included in the plan along with an assumed walking distance of 2 km. The transit network for the region provided by Los Angeles County Metropolitan Transportation Authority is obtained from GTFS Data Exchange.

**Fig 6:**
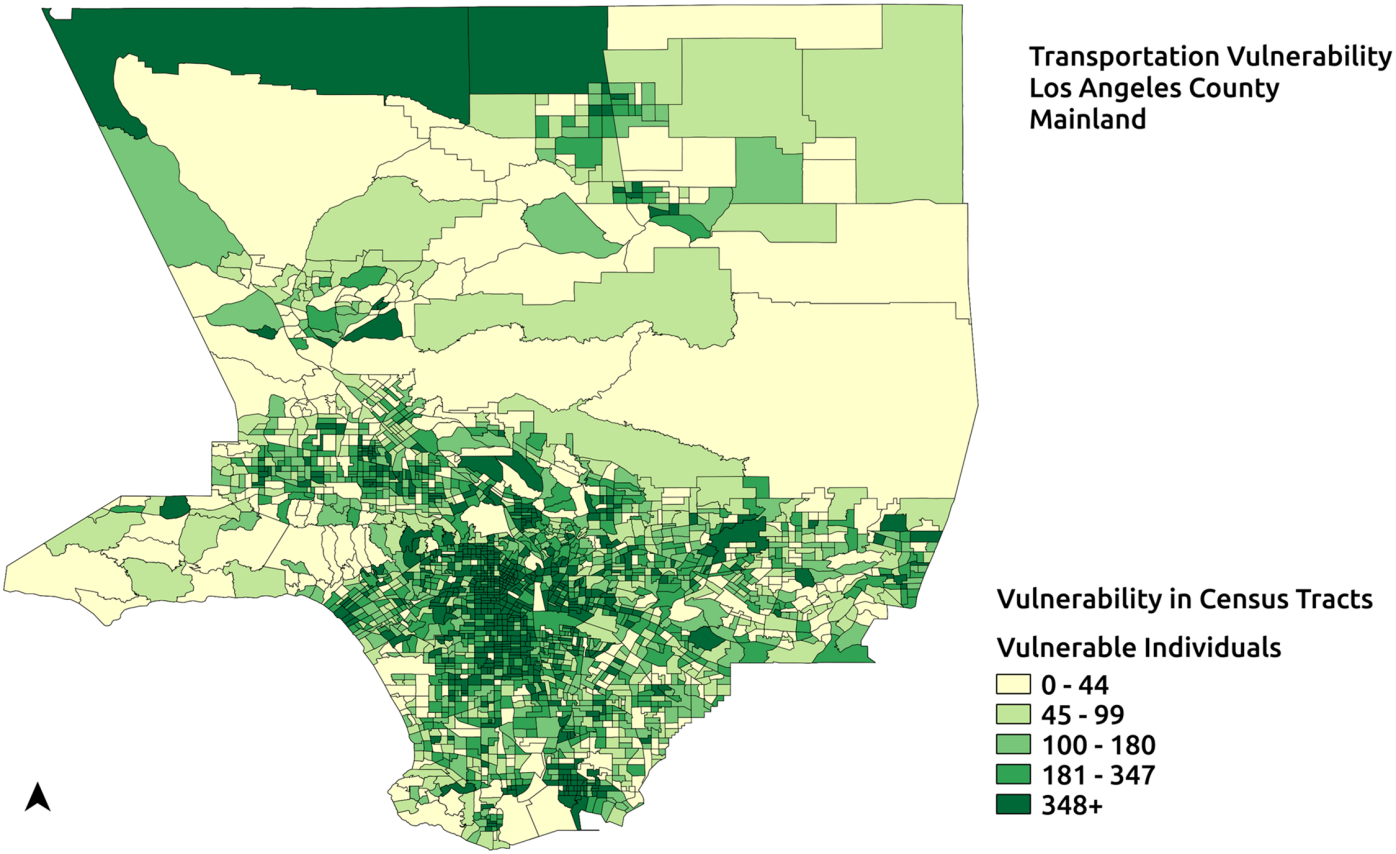
Transportation vulnerable population of the region. Transportation vulnerable population in a potential response plan with 50 service areas.

**Fig 7:**
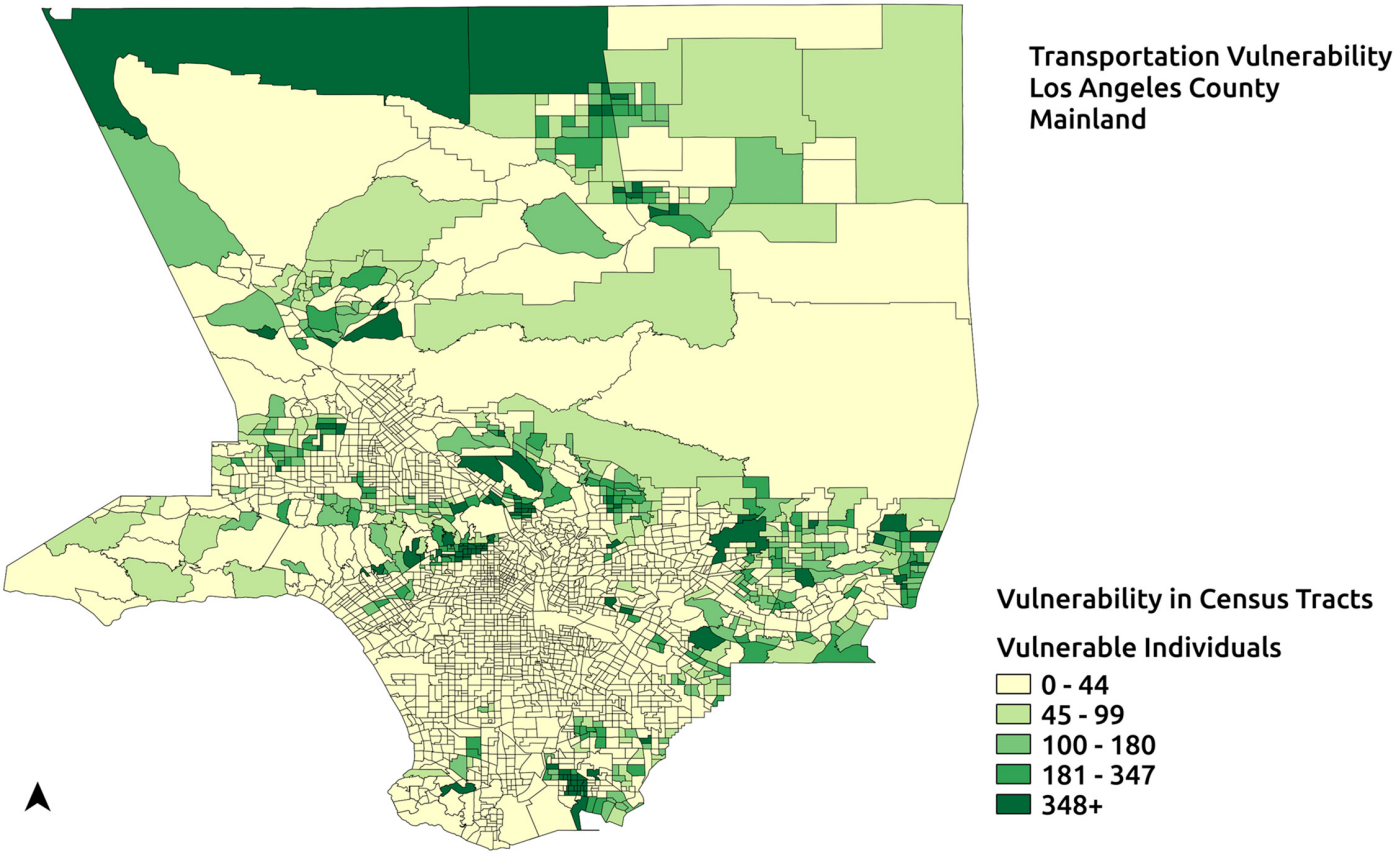
Transportation vulnerable population not served by potential response plan. Vulnerable population after including public transportation and a maximum walking distance of 2 km.

Quantification of transportation vulnerability in the response plan as described earlier reveals that there are 596341 vulnerable individuals who do not have access to private transportation. Fig 6 shows the distribution of vulnerable individuals in the service areas of the response plan. Transportation vulnerable individuals may travel to the PODs by means of public transit, whenever a public transit stop is accessible to them. Transportation vulnerability is quantified after including the Los Angeles County Metropolitan Transportation Authority transit network in the response plan and the maximum walking distance is assumed to be 2 km. Fig 7 depicts the distribution of vulnerable populations still at-risk of not being able to participate in mitigation activities under the assumption of a 2 km maximum walking distance and with the availability of normal public transit operations. In this case, 469705 individuals out of the 596341 vulnerable individuals have access to PODs. This indicates that 126636 individuals in the region access to PODs under the assumptions stated.

### Type-2 Results

The PODs in each of the 50 service areas (Fig 6) generated for the region of study serve approximately equal numbers of individuals. This in turn may imply an approximately identical allocation of resources to PODs. However, such an allocation may not be accurate when considering resources allocated to serve vulnerable populations with specific needs. Language vulnerability is used to demonstrate the access disparities resulting from Type-2 vulnerabilities. Language vulnerability in this case, refers to the sub-population representing the speakers of a specific language who speak English “not well” or “not at all”. They may need additional specific resources to address their needs. For instance, Spanish language vulnerability refers to Spanish speakers who cannot speak English at all or cannot speak English well.

**Fig 8:**
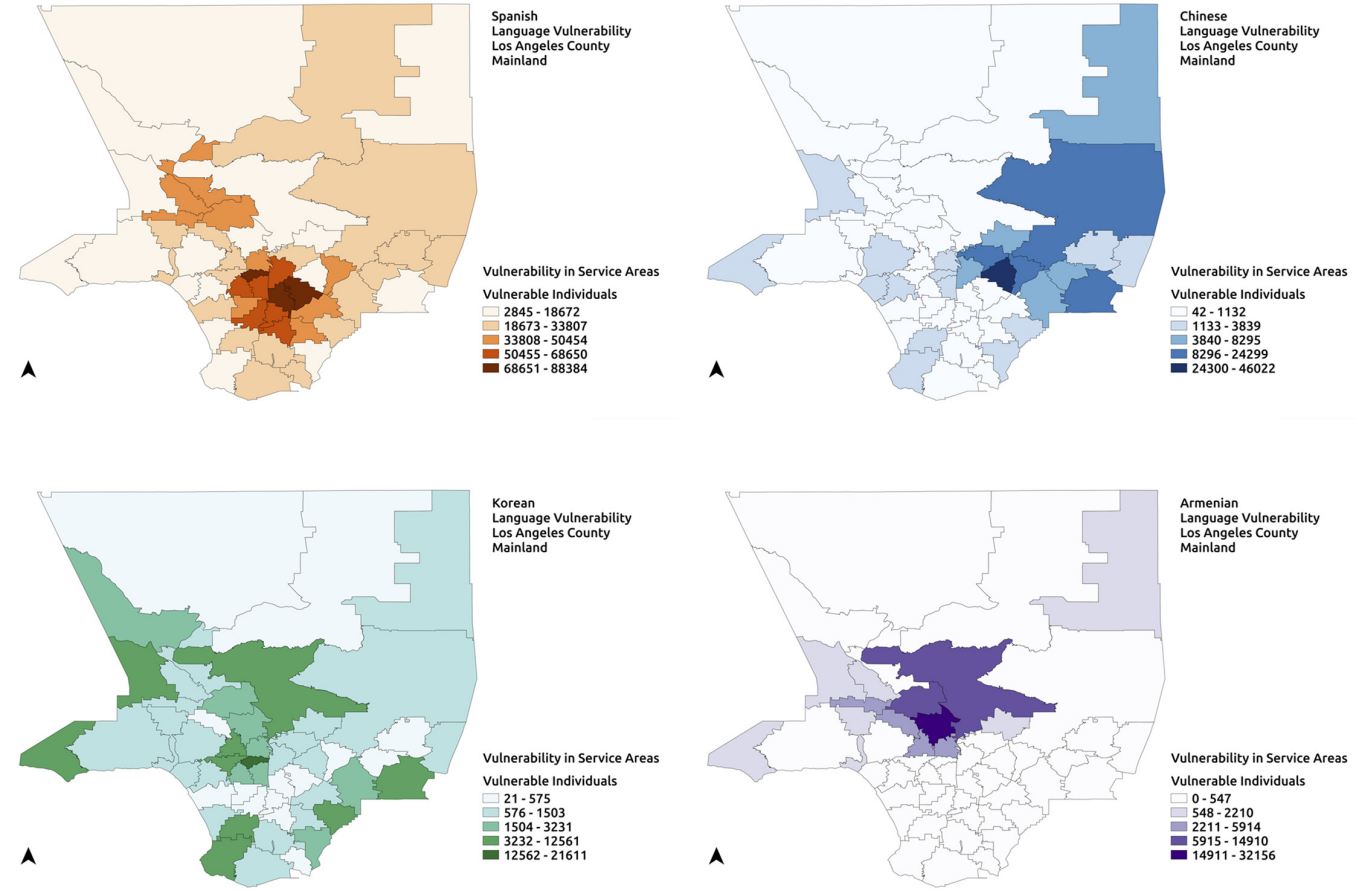
Type-2 language vulnerability quantification. Quantification of Type-2 language vulnerabilities in the response plan developed for the region. (a) Spanish language vulnerability (b) Chinese language vulnerability (c) Korean language vulnerability (d) Armenian language vulnerability.

An analysis of the service areas generated by EPP is performed to demonstrate the imbalances in vulnerable populations across the uniformly populated service areas. Language vulnerabilities for the four most widely spoken languages other than English in this region: Spanish, Chinese, Korean and Armenian languages, are quantified for the response plan generated for the region. Fig 8 shows the language vulnerability for Spanish, Chinese, Korean and Armenian languages in the service areas of the region in relation to the approximately equal populations of the service areas. Variation in language vulnerability at service areas for the different languages chosen can be seen in the maps of the region in Fig 8.

**Fig 9:**
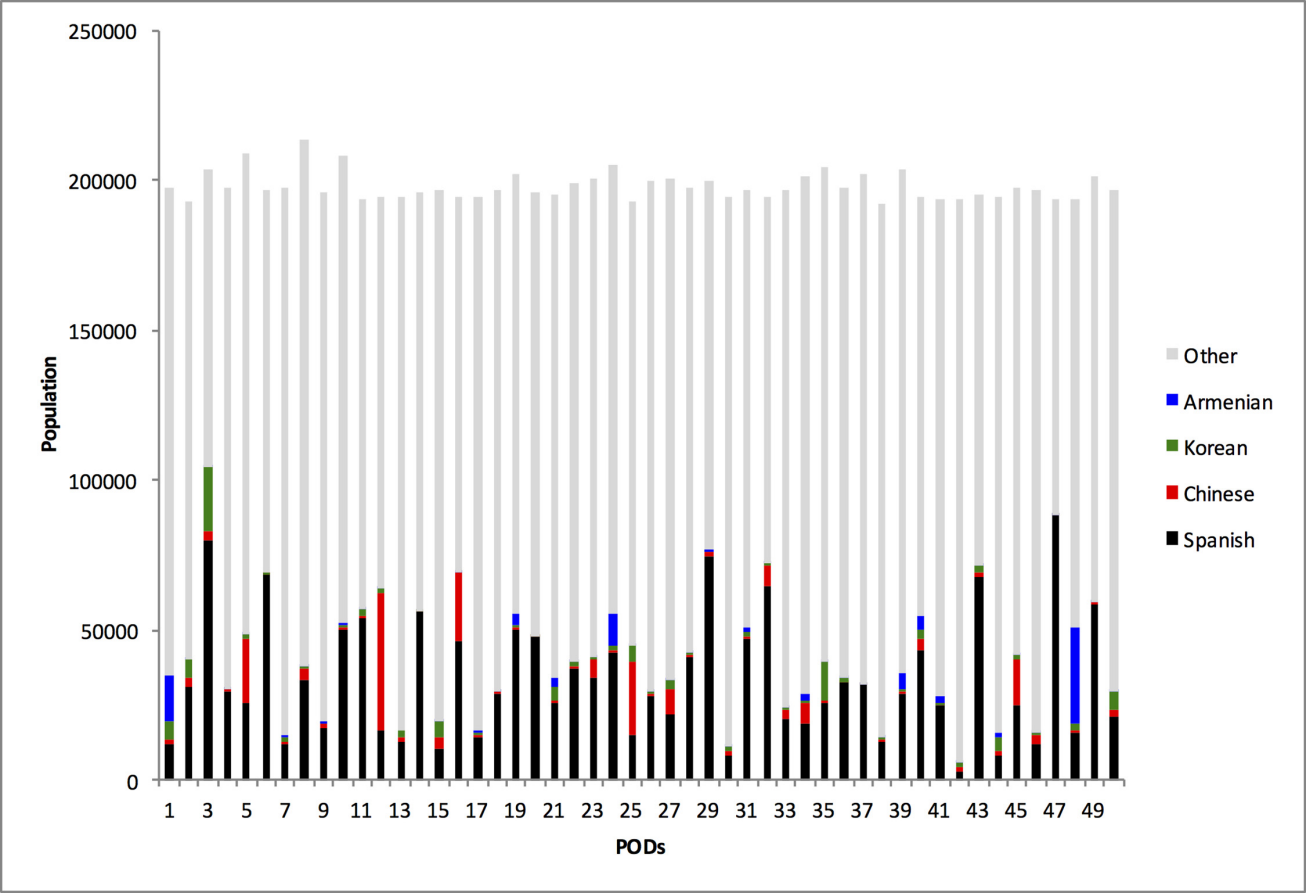
Language Vulnerability. Vulnerable Chinese, Korean, Spanish and Armenian sub-populations in each service area compared to the total population in the service area.

The language vulnerabilities may require specific targeted resources such as translators at the POD. Variations in vulnerabilities within service areas, as can be seen in Fig 9, suggest an allocation that meets local requirements. Further, differences in the numbers of translators located within a service area and the local requirement indicate moving translators between service areas. In such a scenario, it may be of value to minimize the distance traveled by translators to their assigned PODs. Such imbalances necessitate methods to gauge demand for the various resources that may be required during an emergency at individual PODs, and devise methods to determine appropriate allocations.

## Discussion

The quantification of Type-1 and Type-2 vulnerabilities in existing response plans highlights the need to devise specific methodologies to address the access disparities that may have been inadvertently introduced to existing response plans. Type-1 vulnerabilities can be addressed by maximizing the reach of transportation vulnerable populations by the PODs. The effects of Type-2 vulnerabilities can be minimized by the targeted allocation of resources to PODs.

Reach of transportation vulnerable populations by the PODs must be maximized without modifying the existing POD service areas by choosing the best POD location from a set of available alternate locations. Selecting the POD location that yields the greatest reach of vulnerable populations will also maximize the overall reach. Therefore, the reach of vulnerable populations by each of the potential POD locations of a service area must be computed and compared.

Populations which are neither within walking distance of a POD nor a public transit stop must be addressed by strategically modifying the public transit system to reach these populations. Further, the demand-capacity ratio resulting from implementation of response plans must be analyzed for each link in the public transit system to ensure sufficient transit infrastructure is provided. Effectively eliminating the distance between PODs and transit stops by implementing shuttles between them would maximize the coverage areas of PODs via the public transit system.

Type-2 vulnerabilities require the allocation of specific resources at PODs to minimize the consequential access disparities. A response plan designed by establishing a uniform POD design and approximately equally populated service areas may however require an allocation of resources proportional to the POD-wise resource need identified. The availability of resources, either human or supplies, may be limited during an emergency and the resources may have to be sourced from different locations. This necessitates developing methodologies for the optimal allocation of resources to PODs such that the resources are efficiently utilized to minimize the vulnerable population not addressed. The allocation of resources must in addition consider the minimization of distance between the PODs and locations from where the resources are sourced to improve the effectiveness of the plan. Resources may have to be shared between PODs due to constraints with respect to temporal availability or scarcity, which necessitates scheduling of resources. Further, the same resource may be capable of addressing multiple vulnerabilities. For instance, a nurse may also possess the necessary skills and serve as a Spanish language translator during an emergency. Allocating such a resource to a POD with a presence of Spanish language vulnerable populations may be a better utilization of the resource. The methodology developed for the targeted allocation and scheduling of resources to PODs should consider matching the skills of resources to the requirement at PODs in addition to minimizing vulnerable populations not addressed and displacement of resources. The general resource allocation problems are known to be computationally hard [40]. The diversity in types of resources and requirements at PODs clubbed with constraints with respect to availability further complicate the problem of targeted allocation of resources to minimize access disparities, emphasizing on the need to develop computational methodology to devise feasible resource allocation solutions.

## Summary

Vulnerabilities that are a consequence of language, literacy, medical conditions and disabilities, isolation (cultural, geographic, or social), and age may impede access to the assigned PODs during an emergency. Minimizing Access Disparities in a response plan is critical to the effectiveness of the plan. This paper focuses on quantifying the access disparities that could have inadvertently been introduced to the response planning process owing to Type-1 and Type-2 vulnerabilities. Type-1 vulnerabilities involve changes in the placement (location) of infrastructural components, while Type-2 vulnerabilities require adjustment of resource allocation to minimize their effects. Methods to quantify Type-1 and Type-2 vulnerabilities in existing and otherwise feasible response plans are presented in this paper. Lack of access to private transportation, which hinders the ability to reach a POD, exemplifies Type-1 vulnerability. It can be minimized by providing means of transportation to vulnerable populations or by adjusting the placement of PODs to enhance their reach. Type-2 vulnerabilities, demonstrated by language vulnerability, involve population groups that have special needs and require targeted resources at PODs to address them. Quantification of transportation and language vulnerabilities in response plans based on uniform service areas for the region representing the mainland of Los Angeles County, California is demonstrated. By identifying and quantifying the vulnerabilities, measures can be taken to address the access disparities at PODs arising as a consequence of them.

The computational methods described above have been integrated into the RE-PLAN Framework which has been deployed for practical use at state and county offices [7]. Collaborations with practitioners have proven instrumental for understanding planning constraints, capabilities, and practices. To facilitate acceptance of computational methods in the planning process, it is essential that practitioners without GIS or computer programming expertise be able to use planning software. Broad adoption of computational methods by federal and state agencies will result in a standardized planning workflow, thus facilitating data sharing and collaboration among planners at multiple levels of responsibility.

## Supporting Information

S1 Dataset Los Angeles County Response Plan: Language Vulnerability Classes. (TXT)

S2 Dataset Los Angeles County Response Plan: Block Groups to PODs Assignment. (TXT)

S3 Dataset Los Angeles County Response Plan: Service Area Geometries. (TXT)

S4 Dataset Los Angeles County Response Plan: POD Details. (TXT)
